# First-line treatment patterns and lipid target levels attainment in very high cardiovascular risk outpatients

**DOI:** 10.1186/1476-511X-12-170

**Published:** 2013-11-09

**Authors:** Ioanna Xanthopoulou, Periklis Davlouros, Simos Siahos, Angelos Perperis, Evangelia Zaharioglou, Dimitrios Alexopoulos

**Affiliations:** 1 From the Department of Cardiology, Patras University Hospital, Patras, Rion 26500, Greece

**Keywords:** Statin, LDL-C target attainment, Non-HDL-C target attainment, Very high cardiovascular risk

## Abstract

**Objectives:**

Previous studies have demonstrated gaps in achievement of low-density lipoprotein-cholesterol (LDL-C) goals among patients at very high cardiovascular risk. We aimed to investigate lipid treatment patterns, rates and predictors of lipid targets attainment, in such outpatients in an urban area of Greece.

**Methods:**

This was a prospective observational study, conducted in 19 outpatient clinics of Western Greece. We recruited patients with established cardiovascular disease (CVD) and/or diabetes mellitus (DM), previously (at least 3 months before baseline assessment) untreated with any lipid lowering medication. Lipid profile assessment was performed at baseline (prior to lipid-lowering treatment initiation) and at follow-up. Lipid lowering treatment choice was at physicians’ discretion and was kept constant until follow-up.

**Results:**

We recruited 712 patients with a mean age 61.4 ± 10.4 years, 68.0% males, 43.0% with DM, 64.7% with prior coronary artery disease-CAD. In total, 237/712 (33.3%) of prescribed regimens were of high or very high LDL-C lowering efficacy and out of them 113/237 (47.7%) comprised a combination of statin and ezetimibe. At follow-up the primary target of LDL-C < 70 mg/dL (1.8 mmol/L) was achieved in 71(10.0%) patients. The secondary target of non-HDL-C < 100 mg/dL (2.6 mmol/L) in the subgroup of patients with DM or increased triglycerides levels (>150 mg/dl or 1.7 mmol/L) was achieved in 45(11.6%) of patients. In multivariate logistic regression analysis (AUC = 0.71, 95% CIs 0.65-0.77, p < 0.001) male gender, smoking, baseline LDL-C and very high potency LDL-C lowering regimen emerged as independent predictors of LDL-C goal attainment (OR = 1.88, 95% CIs 1.03-3.44, p = 0.04, OR = 0.57, 95% CIs 0.33-0.96, p = 0.04, OR = 0.98, 95% CIs 0.98-0.99, p < 0.001 and OR = 2.21, 95% CIs 1.15-4.24, p = 0.02 respectively).

**Conclusions:**

First-line management of dyslipidemia among very-high cardiovascular risk outpatients in Western Greece is unsatisfactory, with the majority of treated individuals failing to attain the LDL-C and non-HDL-C targets. This finding points out the need for intensification of statin treatment in such patients.

## Introduction

Cardiovascular disease (CVD) is the leading cause of global mortality, accounting for more deaths annually than any other cause [1]. Modification of the risk factors related to CVD, such as dyslipidemia, smoking, or a sedentary lifestyle, has been shown to reduce CVD mortality and morbidity [2].

It has been demonstrated that low density lipoprotein-cholesterol (LDL-C) reduction by statins substantially reduces cardiovascular morbidity and mortality in both primary and secondary prevention [3-5]. In 2004 the National Cholesterol Education Program Adult Treatment Panel III (NCEP-ATP III) guidelines were updated with the addition of the optional goal of LDL-C < 70 mg/dL (1.8 mmol/L) for those patients considered to be at very high cardiovascular risk, mainly based on evidence from Heart Protection Study (HPS) and The Pravastatin or Atorvastatin Evaluation and Infection Therapy (PROVE IT) trials [6-8]. Subsequently, Incremental Increase in End Points Through Aggressive Lipid Lowering (IDEAL) and Treating to New Targets (TNT) studies provided evidence that more intensive versus moderate LDL-C lowering treatment reduces the risk of major cardiovascular events in patients with coronary artery disease (CAD) [9,10]. A recent meta-analysis of several clinical trials involving >170,000 patients revealed a dose-dependent reduction in CVD morbidity and mortality with LDL-C reduction [3]. Accumulating evidence with respect to the beneficial effect on clinical outcome of intensified statin therapy led the 2011 European Society of Cardiology (ESC)/European Atherosclerosis Society (EAS) guidelines to adopt LDL-C < 70 mg/dL (1.8 mmol/L) as the main treatment goal in the subgroup of patients considered to be at very high cardiovascular risk [2].

However, despite cumulating data from large clinical trials demonstrating the benefit of LDL-C goal achievement, which is recommended by the guidelines, a significant treatment gap still remains, especially in patients considered at very high cardiovascular risk: National Cholesterol Education Program Evaluation ProjecT Utilizing Novel E-Technology (NEPTUNE) and Lipid Treatment Assessment Project 2 (L-TAP 2) trials, which were conducted between 2003 and 2007, showed that only 17.8%-34% of very high risk patients attained the optional goal of LDL-C < 70 mg/dL (1.8 mmol/L) [11,12]. It would be interesting to assess lipid lowering management after the release of the 2011 ESC/EAS guidelines.

The present study aimed to investigate lipid-lowering drug therapy used in every-day clinical practice, rates and predictors of treatment target attainment among outpatients with established CVD and/or diabetes mellitus (DM), considered to be at very-high cardiovascular risk in an urban area of Greece.

## Methods

This was a prospective, observational study, conducted between June 2011 and January 2012, in the county of Achaia, Western Greece (327,316 inhabitants in 2001), in 17 private and 2 public (1 tertiary and 1 regional hospital) outpatient cardiology clinics. We identified patients with established CVD and/or DM, previously (at least 3 months before baseline assessment) untreated with any lipid lowering medication.

Patients with established CVD were considered those with known CAD or non-coronary forms of atherosclerotic disease: peripheral arterial disease (PAD), abdominal aortic aneurysm, cerebrovascular disease (ischemic stroke/transient ischemic attack or >50% obstruction of a carotid artery on ultrasound). CAD was defined as any of the following: prior myocardial infarction (MI), prior percutaneous coronary intervention (PCI) or coronary artery by-pass grafting (CABG), documented unstable angina, positive non-invasive stress testing (nuclear imaging or stress echocardiography) or ≥50% stenosis of at least one major coronary artery on angiography. Presence of PAD was determined by a history of intermittent claudication, decreased pulses or bruit with ankle-brachial index <0.90, or abnormal duplex ultrasound. DM was defined as pre-existing diagnosis of DM made by a physician, use of oral-hypoglycaemic agents or insulin or 2 fasting glucose measurements >126 mg/dL [13].

Lipid profile assessment was performed at baseline (prior to initiation of any lipid-lowering treatment) and at follow-up. Data extracted from patient’s medical records were collected and a case report form was completed. Only patients with complete lipid profile data [total cholesterol (TC), LDL-C, high-density lipoprotein-cholesterol (HDL-C) and triglycedides (TG)] at baseline and at follow-up were included in analyses. Lipid lowering treatment choice as well as lipid level monitoring and response to treatment was at clinicians’ discretion. Lipid-lowering treatment was kept constant until lipid profile assessment at follow-up and patients with treatment changes were excluded from analysis. Lipid assessment was performed in commercial laboratories, in the context of usual clinical practice, under fasting conditions. HDL-C was measured directly in the serum. Friedewald formula was used for LDL-C calculation [14]. Non-HDL-C was calculated as TC minus HDL-C. Patients with known familial hypercholesterolemia or TG > 400 mg/dL, were excluded from the present analysis.

Primary treatment target was LDL-C < 70 mg/dL (1.8 mmol/L), as the studied population was considered to be at very high cardiovascular risk. Reiner Z, 2011 [2] Non-HDL-C < 100 mg/dL (2.6 mmol/L) was a secondary treatment target, in the subgroup of patients with DM or increased TG levels (>150 mg/dL or 1.7 mmol/L) [2].

Lipid lowering regimens were classified by their % LDL-C reduction efficacy, according to dose and kind of statin used and whether ezetimibe was prescribed as an adjunct (combined therapy with ezetimibe and a statin was considered to provide an incremental reduction in LDL-C of 15%). As shown in Table 1, four groups of lipid lowering regimens were identified: low, moderate, high and very high-efficacy group, which included regimens producing ≤30%, 31-45%, 46-55% and >55% LDL-C reduction respectively [2]. Lipid lowering regimens with at least low, moderate, high, or very high-efficacy were considered as an appropriate treatment selection to achieve the goal of LDL-C <70 mg/dL (1.8 mmol/L) at follow-up, in patients with baseline LDL-C <100, 100–130, 131–150 and >150 mg/dL respectively.

**Table 1:** Classification of lipid lowering regimens used by their LDL-C lowering efficacy

	**Low % LDL-C reduction**	**Moderate % LDL-C reduction**	**High % LDL-C reduction**	**Very high % LDL-C reduction**
**Simvastatin**	10 mg	10 mg + Eze		40 mg + Eze
		20 mg	80 mg	
			20 mg + Eze	80 mg + Eze
		40 mg		
**Fluvastatin**	40 mg	80 mg		
			80 mg + Eze	
**Rosuvastatin**		5 mg	20 mg	5 mg + Eze
				10 mg + Eze
			10 mg	20 mg + Eze
				40 mg
				40 mg + E
**Pravastatin**	20 mg	20 mg + Eze		
	40 mg	40 mg + Eze		
		80 mg		
**Atorvastatin**		10 mg		20 mg + Eze
		20 mg	40 mg	40 mg + Eze
		20 mg	10 mg + Eze	
**Ezetimibe**	10 mg			
**Fenofibrate**	200 mg			

Eze = ezetimibe.

Study’s protocol was approved by Patras University Hospital Ethics Committee and all participants gave written informed consent.

### Statistical analysis

Categorical data are presented as frequencies and group percentages and continuous data as means ± standard deviation. Two-sample t-test and the Fisher’s exact test were used for comparison of continuous and categorical data respectively. Multivariate logistic regression analysis (in a backward elimination fashion, p > 0.1 for removal) was used to assess independent predictors of LDL-C goal attainment at follow-up, controlling for age, gender, diabetes mellitus, presence of CAD, PAD, cerebrovascular disease, smoking at baseline assessment, baseline LDL-C, combined therapy with ezetimibe and a statin and very high potency LDL-C lowering regimen. Fenofibrate and n3 fatty acids use were additionally investigated as potential predictive factors of non-HDL-C goal attainment. Models were tested for discriminative power by the C statistic [area under the receiver operating characteristic (ROC) curve]. All tests were 2-tailed and statistical significance was considered for p-values < 0.05. Statistical analyses were performed using SPSS for Windows (version 16.0 SPSS Inc.Chicago II USA).

## Results

### Study population

**Figure 1:**
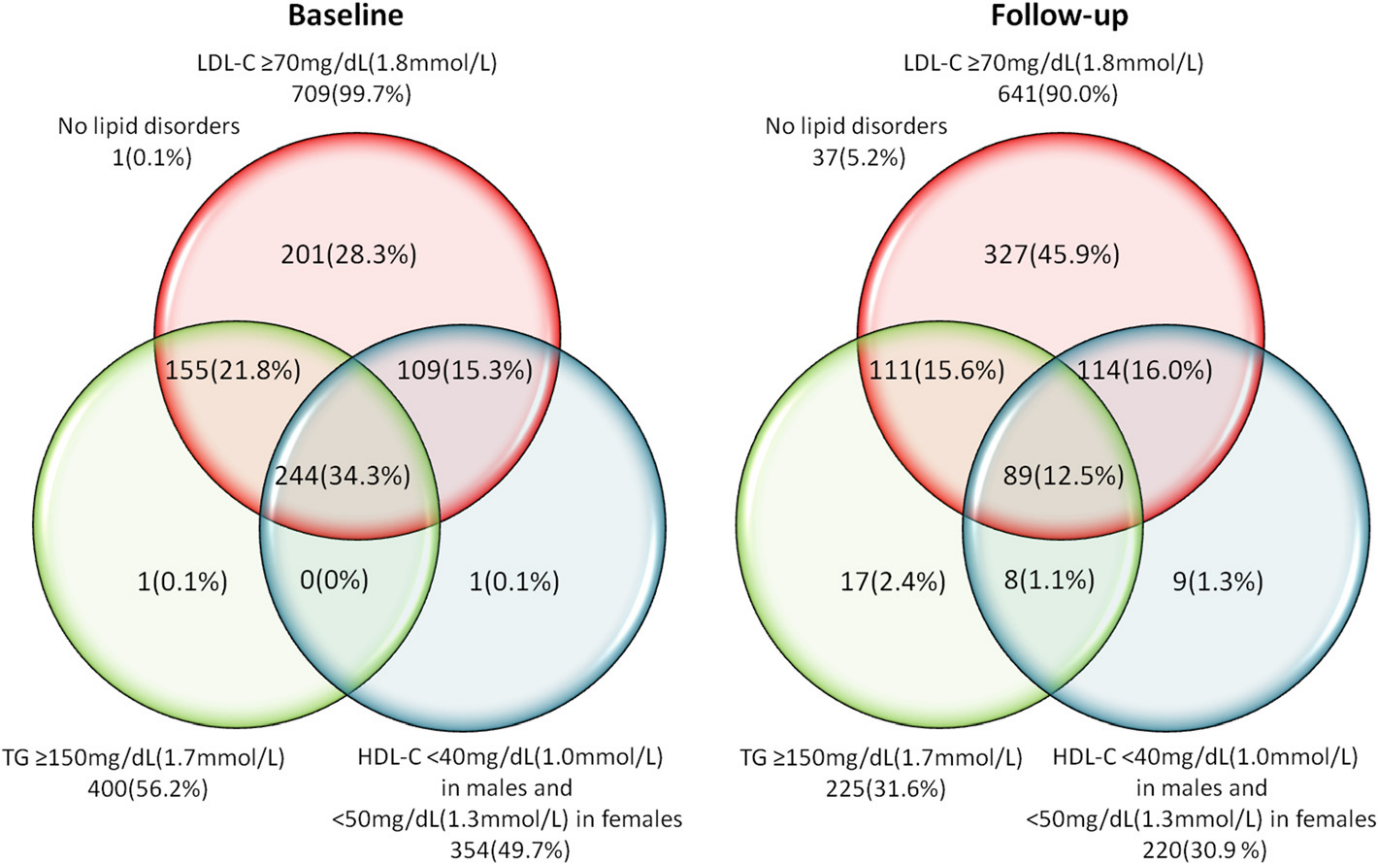
Lipid disorders rates before initiation of lipid modifying treatment and at follow-up.

The study population consisted of 712 outpatients (mean age 61.4 ± 10.4 years, 68.0% males). Patients’ baseline characteristics and lipid values by LDL-C goal attainment at follow-up are depicted in Table 2. Lipid profile assessment at follow-up was performed at a median time of 3.0 (2.9-3.3 first to third quartile) months. The majority (64.7%) of patients had prior CAD, 43.0% of them were diabetics and 54.1% were current smokers, with a mean LDL-C of 167.0 ± 35.6 mg/dL at time of baseline assessment. As shown in Figure 1, at baseline only 1(0.1%) patient had no lipid disorders, whereas 709(99.7%) patients had LDL-C ≥70 mg/dL (1.8 mmol/L).

**Table 2:** Baseline characteristics of study population

	**Overall N = 712**	**LDL-C goal attainment N = 71**	**No LDL-C goal attainment N = 641**	**p-value**
Age (years)	61.4 ± 10.4	63.2 ± 11.3	61.2 ± 10.3	0.1
Male gender	484 (68.0)	55 (77.5)	429 (66.9)	0.08
Cardiovascular risk factors				
Prior CAD	461 (64.7)	53 (74.6)	408 (63.7)	0.07
Cerebrovascular disease	64 (9.0)	1 (1.4)	63 (9.8)	0.01
Abdominal aortic aneurysm	24 (3.4)	8 (11.3)	55 (8.6)	0.5
Peripheral arterial disease	63 (8.8)	8 (11.3)	55 (8.6)	0.5
Hypertension	458 (64.3)	44 (62.0)	414 (64.6)	0.7
Smoking	385 (54.1)	29 (40.8)	356 (55.5)	0.02
Diabetes mellitus	306 (43.0)	27 (38.0)	279 (43.5)	0.4
Family history of CAD	164 (23.0)	13 (18.3)	151 (23.6)	0.4
Lipid levels at baseline				
TC (mg/dl)	248.5 ± 37.3	232.7 ± 36.9	250.3 ± 36.9	<0.001
LDL-C (mg/dl)	167.0 ± 35.6	150.1 ± 34.5	168.8 ± 35.3	<0.001
HDL-C (mg/dl)	45.7 ± 11.3	46.6 ± 10.1	45.6 ± 11.4	0.5
TG (mg/dl)	165.2 ± 68.4	170.7 ± 79.8	164.6 ± 67.0	0.5
Non HDL-C (mg/dl)	202.9 ± 37.7	186.1 ± 37.7	204.7 ± 37.3	<0.001

Data are expressed as means ± SD or n (%). CAD = coronary artery disease, TC = total cholesterol, LDL-C = low-density lipoprotein-cholesterol, HDL-C = high-density lipoprotein-cholesterol, TG = triglycerides.

### Lipid lowering treatment patterns

**Figure 2:**
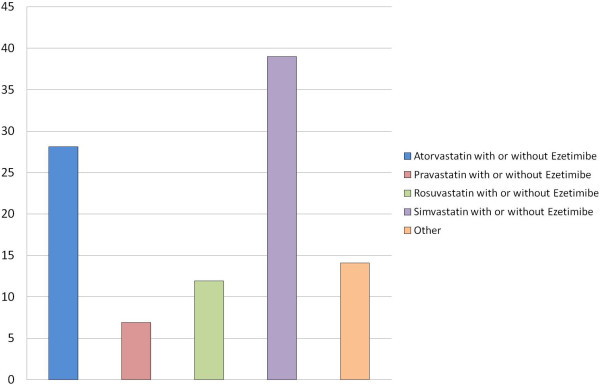
Lipid modifying medications used in the study population, category “other” includes regimens representing <1% of all prescribed treatments.

**Figure 3:**
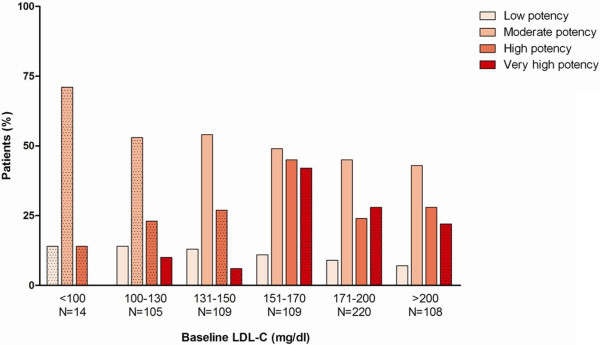
**LDL-C lowering potency of lipid lowering therapy by baseline LDL-C, bars represent percentages within each baseline LDL-C subgroup.** Dotted pattern indicates treatment appropriateness as a function of baseline LDL-C to achieve the target of LDL-C<70 (1.8mmol/L) at follow-up.

Lipid lowering regimens used included the following antilipidemic drug classes: statins, fibrates, ezetimibe and n3 fatty acids. Statin monotherapy was the most frequently prescribed regimen (65.3%) while 29.0% of patients were prescribed a combination of statin and ezetimibe at the baseline visit (Table 3). Simvastatin was the most frequently prescribed statin (Figure 2). The majority of patients received a regimen of moderate LDL-C lowering efficacy overall and in each baseline LDL-C subgroup of patients (Figure 3). In total, only 237/712 (33.3%) of prescribed regimens had appropriate LDL-C lowering efficacy, out of them 113/237(47.7%) comprised a combination of statin and ezetimibe.

**Table 3:** Lipid modifying treatment of study population

	**Overall N = 712**	**LDL-C goal attainment N = 71**	**No LDL-C goal attainment N = 641**	**p-value**
**Monotherapy**	476 (66.9)	43 (60.6)	433 (67.6)	0.2
Statin	465 (65.3)	43 (60.6)	422 (65.8)	0.4
Fibrate	4 (0.6)	0 (0)	4 (0.6)	1.0
Ezetimibe	7 (1.0)	0 (0)	8 (1.2)	1.0
**Combination therapy**	236 (33.1)	28 (39.4)	208 (32.4)	0.2
Statin + Ezetimibe	182 (25.6)	22 (31.0)	160 (25.0)	0.3
Statin + Ezetimibe + n3 fatty acids	24 (3.4)	2 (2.8)	22 (3.4)	1.0
Statin + n3 fatty acids	24 (3.4)	2 (2.8)	22 (3.4)	1.0
Other	6 (0.8)	2 (2.8)	4 (0.6)	0.1
**% LDL-C reduction efficacy**				0.5
low	42 (5.9)	4 (5.6)	38 (5.9)	
moderate	486 (68.3)	45 (63.4)	441 (68.8)	
high	160 (22.5)	18 (25.4)	142 (22.2)	
very high	24 (3.4)	4 (5.6)	20 (3.1)	

Data are expressed as n (%).

### Treatment target attainment at follow-up

At follow-up the primary target of LDL-C < 70 mg/dL (1.8 mmol/L) was achieved in 71(10.0%) patients. In total 389 patients were eligible for the secondary target of non HDL-C, which was achieved in 45(11.6%) of them. Figure 1 shows rates of all lipid disorders at baseline and at follow-up.

### Multivariate analysis

In multivariate logistic regression analysis (AUC = 0.71, 95%CIs 0.65-0.77, p < 0.001) male gender, smoking, baseline LDL-C and very high potency LDL-C lowering regimen emerged as independent predictors of LDL-C goal attainment (OR = 1.88, 95%CIs 1.03-3.44, p = 0.04, OR = 0.57, 95%CIs 0.33-0.96, p = 0.04, OR = 0.98, 95%CIs 0.98-0.99, p < 0.001 and OR = 2.21, 95%CIs 1.15-4.24, p = 0.02 respectively). There was a trend towards lower rates of LDL-C goal attainment in patients with cerebrovascular disease (OR = 0.15, 95%CIs 0.02-1.14, p = 0.07), as seen in Table 4. Baseline LDL-C (OR = 0.98, 95%CIs 0.97-0.99, p < 0.001) emerged as the only independent predictor of non-HDL-C target attainment (AUC = 0.69, 95%CIs 0.62-0.77, p < 0.001), as seen in Table 5.

**Table 4:** Predictors of LDL-C goal attainment

	**Initial model**		**Final model**	
	OR (95% CIs)	P-value	OR (95% CIs)	P-value
Male gender	1.66 (0.88-3.13)	0.1	1.88 (1.03-3.44)	0.04
Age	1.02 (0.99-1.04)	0.3		
DM	0.92 (0.49-1.76)	0.8		
Smoking	0.62 (0.36-1.07)	0.09	0.57 (0.33-0.96)	0.04
CAD	1.34 (0.65-2.77)	0.4		
PAD	1.53 (0.65-3.58)	0.3		
Cerebrovascular disease	0.14 (0.02-1.004)	0.05	0.15 (0.02-1.14)	0.07
Baseline LDL-C (mg/dl)	0.99 (0.98-0.99)	<0.001	0.99 (0.98-0.99)	<0.001
Combined therapy with statin and ezetimibe	1.15 (0.56-2.34)	0.7		
Very high potency LDL-C lowering regimen	1.94 (0.84-4.49)	0.1	2.21(1.15-4.24)	0.02

DM = diabetes mellitus, CAD = coronary artery disease, PAD = peripheral arterial disease, LDL-C = low density lipoprotein-cholesterol.

**Table 5:** Predictors of non HDL-C goal attainment

	**Initial model**		**Final model**	
	OR (95% CIs)	P-value	OR (95% CIs)	P-value
Male gender	1.04 (0.50-2.17)	0.9		
Age	1.01 (0.98-1.05)	0.4		
DM	0.78 (0.33-1.87)	0.6		
Smoking	0.65 (0.31-1.36)	0.2		
CAD	1.61 (0.75-3.45)	0.2	1.80 (0.95-3.42)	0.07
PAD	1.71 (0.62-4.66)	0.3		
Cerebrovascular disease	0.64 (0.17-2.35)	0.5		
Baseline LDL-C (mg/dl)	0.98 (0.97-0.99)	0.001	0.98 (0.97-0.99)	<0.001
Combined therapy with statin and ezetimibe	1.92 (0.81-4.56)	0.1		
Very high potency LDL-C lowering regimen	1.06 (0.36-3.10)	0.9		
Fenofibrate	1.05 (0.34-3.42)	0.9		
n3 fatty acids				

DM = diabetes mellitus, CAD = coronary artery disease, PAD = peripheral arterial disease, LDL-C = low density lipoprotein-cholesterol.

## Discussion

This prospective, observational study provides insights into the lipid lowering treatment of outpatients at very high cardiovascular risk in Western Greece. The main findings of our study are: a) Current first-line management of dyslipidemia seems to be unsatisfactory, since most of the treated individuals failed to attain the LDL-C target at follow-up b) In the majority of patients, prescribed lipid lowering regimens had a suboptimal LDL-C % reduction efficacy, as a function of baseline LDL-C, to attain the goal of <70 mg/dl (1.8 mmol/L). c) Approximately 1/3 of patients were prescribed a combination of statin and ezetimibe as first-line lipid-lowering treatment. Our study points out a significant therapeutic gap in treatment of patients at very high cardiovascular risk, mainly due to prescription of suboptimal lipid lowering regimens.

Based on several randomized clinical trials [3,9,10], current ESC guidelines recommend LDL-C < 70 mg/dL (1.8 mmol/L) as the primary treatment target in patients at very high cardiovascular risk [2]. However, observational studies show that only a small proportion of patients with CAD achieve this goal [15-18]. A survey which included longitudinal data from 18,656 participants, showed that in U.S. achievement of LDL-C < 70 mg/dL (1.8 mmol/L) improved from 2.4% to 17% (p < 0.0001) in high-risk individuals and patients with CAD (3.4% to 21.4%, p < 0.0001) over the last decade, but still remains insatisfactory [19]. Our findings are in the same line of evidence, with the vast majority of patients failing to attain LDL-C treatment target.

Several reasons may contribute to this observed treatment gap. First, underestimation of cardiovascular risk by the treating physician could have resulted in a poor LDL-C target choice, leading to a lipid-lowering treatment of suboptimal efficacy. As shown by Sager et al. in a study which evaluated 907 physicians’ perception of guideline-recommended low-density lipoprotein target values, approximately 50% of high-risk patients did not receive correct assignment of LDL target by their physicians [20]. In addition, lack of awareness of the new, more aggressive target of LDL-C < 70 mg/dL (1.8 mmol/L) by the treating physicians, could have lead to the suboptimal management of dyslipidemia in these patients [21]. However, failure to initiate a lipid lowering treatment of appropriate efficacy is probably the main reason for the observed low treatment goal attainment in our population. Indeed, only 33.3% of patients were prescribed a lipid lowering regimen of appropriate efficacy, finding is in accordance with other observational studies [20-22].

It is of note that in our study, a significant proportion of patients did not receive statin monotherapy but a combination of statin and ezetimibe as starting treatment. This can be explained by reluctance of physicians to prescribe statins at high doses by the fear of side effects like myotoxicity [22] and by evidence supporting a stronger LDL-C lowering effect of combination therapy with statin and ezetimibe versus statin monotherapy [23-26]. Statin treatment is in general considered safe and serious adverse events are rare. However, mainly in individuals with comorbidities, statin treatment may lead to myopathy, a potentially fatal condition [2,22]. Statins may also lead to transaminase elevation, which is dose-dependent and usually reversible by reduction of dose [2]. Ezetimibe provides when combined with a statin an incremental reduction in LDL-C levels of 15-20% and can be used as a combination with statins, in patients with poor statin tolerance [2].

Male gender and treatment with very high potency LDL-C lowering regimen favored primary treatment target attainment, consistently with previous reports [17,27,28]. In addition, our multivariate analysis showed that current smokers and those with higher baseline LDL-C were less likely to achieve LDL-C target, also in accordance with other studies [11,17,21,29].

Furthermore, our study showed that success rate for the secondary target of non-HDL-C was also very low. Evaluation of success in attaining non-HDL-C goal in the multinational L-TAP 2 study revealed a 34% success rate among patients at very high cardiovascular risk, however among those patients who failed to attain their LDL-C target in the overall population only 11.2% achieved the non-HDL-C target [12]. Therefore, the very low success rate of non-HDL-C observed in our study might be explained by the poor LDL-C goal achievement.

The low treatment goal attainment among patients at very high cardiovascular risk, underscores the need to improve management of dyslipidaemia in those patients. Treatment intensification with selection of appropriate dose and drug as first-line treatment as well as dose titration when needed will help translating the important benefits that statins demonstrated in large-scale clinical trials to the real world.

Our study underscores the need to use a statin (at the appropriate dose) as first-line treatment of dyslipidemia. Otherwise, suboptimal dosing may lead to a high proportion of patients not achieving the LDL-C goal. These therapeutic options are clearly highlighted in the 2011 ESC/EAS guidelines, which recommend that a statin should be the first-line pharmacological treatment of dyslipidaemia, prescribed at a dose capable to provide the percentage LDL-C reduction to achieve the LDL-C target for patient’s individual CV risk level. If LDL-C target is not achieved after dose titration, statin combination with bile acid sequestrant, nicotinic acid or cholesterol absorption inhibitor may be considered [2]. In addition, the ongoing large outcome trials: Improved Reduction of Outcomes: Vytorin Efficacy International Trial (IMPROVE-IT) and Efficacy and Safety of Alirocumab SAR236553 (REGN727) Versus Placebo on Top of Lipid-Modifying Therapy in Patients With High Cardiovascular Risk and Hypercholesterolemia (ODYSSEY Combo I) will provide important information on the role of combined lipid-lowering therapy in the treatment of patients at high cardiovascular risk.

Several limitations apply to our study. As we only studied patients managed by a cardiologist our findings may not reflect the practices of other physicians. Furthermore, our results may underestimate the prevalence of lipid-lowering treatment failure in the general population, as selection of participating physicians and patients was based on their consent and complete lipid profile availability. In addition, as our study was conducted shortly after the release of 2011 ESC/EAS guidelines, we cannot exclude the existence of potential temporal trend in perception of guidelines and management of dyslipidaemias by the treating physicians. Allowing a longer interval before recheck lipid levels is probably considered as an “off-label” practice. However, no evidence based information exists and low rates of goal attainment at follow-up, could certainly not be attributed to inadequate treatment length. We did not use a central laboratory for lipid measurements; however this rather reflects the real-life clinical practice. Finally, adherence to treatment was not monitored.

## Conclusions

First-line management of dyslipidemia among very-high cardiovascular risk outpatients in Western Greece is unsatisfactory, with the majority of treated individuals failing to attain the LDL-C and non-HDL-C targets. This observed treatment gap although disappointing, translates into an opportunity to ameliorate clinical outcome of such patients. Therefore, strategies to improve management of dyslipidemia, such as intensification of first-line statin treatment, are necessary.

## Competing interests

Dr Alexopoulos reports receipt of speakerfees from AstraZeneca. This study was supported by the Research Committee of the Patras University Medical School.

## Authors’ contributions

Analyzed, interpreted the data and drafted manuscripts: IX, PD, SS, AP and ZE, conception, design and final approval of the manuscript DA. All authors read and approved the final manuscript.
